# HIV Research for Prevention 2018: From Research to Impact Conference Summary and Highlights

**DOI:** 10.1089/aid.2019.0074

**Published:** 2019-06-26

**Authors:** Barbara L. Shacklett, Julià Blanco, Lisa Hightow-Weidman, Nyaradzo Mgodi, José Alcamí, Susan Buchbinder, Mike Chirenje, Smritee Dabee, Mamadou Diallo, Kostyantyn Dumchev, Carolina Herrera, Matthew E. Levy, Enrique Martin Gayo, Nigel Aminake Makoah, Kate M. Mitchell, Kenneth Mugwanya, Krishnaveni Reddy, Maria Luisa Rodríguez, Marta Rodriguez-Garcia, Chelsea L. Shover, Tripti Shrivastava, Georgia Tomaras, Michiel Van Diepen, Monika Walia, Mitchell Warren, Amapola Manrique, Bargavi Thyagarajan, Tamara Torri

**Affiliations:** ^1^ Medical Microbiology and Immunology, University of California, Davis, Davis, California.; ^2^ IrsiCaixa, IGTP, Barcelona, Spain.; ^3^ Chair in AIDS and Related Illnesses, Centre for Health and Social Care Research (CESS), Faculty of Medicine, University of Vic-UCC, Barcelona, Spain.; ^4^ Division of Infectious Diseases, University of North Carolina, Chapel Hill, Chapel Hill, North Carolina.; ^5^ University of Zimbabwe College of Health Sciences, Harare, Zimbabwe.; ^6^ AIDS Immunopathology Unit, Instituto de Salud Carlos III, Madrid, Spain.; ^7^ San Francisco Department of Public Health, San Francisco, California.; ^8^ Division of Medical Virology, University of Cape Town, Cape Town, South Africa.; ^9^ Department of Social and Preventive Medicine, University Laval, Quebec, Canada.; ^10^ Research, Ukrainian Institute on Public Health Policy, Kyiv, Ukraine.; ^11^ Faculty of Medicine, Department of Medicine, Imperial College London, London, United Kingdom.; ^12^ Department of Epidemiology and Biostatistics, The George Washington University, Washington, District of Columbia.; ^13^ Immunology Department, Hospital Universitario La Princesa, Universidad Autónoma de Madrid, Madrid, Spain.; ^14^ Centre for HIV and STIs, National Institute for Communicable Diseases, Johannesburg, South Africa.; ^15^ MRC Centre for Global Infectious Disease Analysis, Imperial College London, London, United Kingdom.; ^16^ HPTN Modelling Centre, Imperial College London, London, United Kingdom.; ^17^ Department of Epidemiology, University of Washington, Seattle, Washington.; ^18^ Wits Reproductive Health and HIV Institute, University of the Witwatersrand, School of Clinical Medicine, Johannesburg, South Africa.; ^19^ Department of Physiology and Neurobiology, Geisel School of Medicine at Dartmouth, Lebanon, New Hampshire.; ^20^ Department of Psychiatry and Behavioral Sciences, Stanford University, Palo Alto, California.; ^21^ Department of Infection and Immunology, Translational Health Science and Technology Institute, Faridabad, India.; ^22^ Departments of Surgery, Immunology, and Molecular Genetics and Microbiology, Duke University, Durham, North Carolina.; ^23^ Population Council, New York, New York.; ^24^ AVAC, New York, New York.; ^25^ Global HIV Vaccine Enterprise, New York, New York.; ^26^ International AIDS Society, Geneva, Switzerland.

**Keywords:** clinical trial, bNAbs, HIVR4P, immunogens, TasP, Env

## Abstract

The HIV Research for Prevention (HIVR4P) conference is dedicated to advancing HIV prevention research, responding to a growing consensus that effective and durable prevention will require a combination of approaches as well as unprecedented collaboration among scientists, practitioners, and community workers from different fields and geographic areas. The conference theme in 2018, “From Research to Impact,” acknowledged an increasing focus on translation of promising research findings into practical, accessible, and affordable HIV prevention options for those who need them worldwide. HIVR4P 2018 was held in Madrid, Spain, on 21–25 October, with >1,400 participants from 52 countries around the globe, representing all aspects of HIV prevention research and implementation. The program included 137 oral and 610 poster presentations. This article presents a brief summary of highlights from the conference. More detailed information, complete abstracts as well as webcasts and daily Rapporteur summaries may be found on the conference website.

## Introduction

Opening plenary talks, delivered by **Linda-Gail Bekker** (Abstract PL01.01)^1^ (recipient of the 2018 Desmond Tutu Award for HIV Prevention Research and Human Rights), **Anthony Fauci** (Abstract PL01.03),^1^ and **Sheena McCormack** (Abstract PL01.02),^1^ provided important perspectives on the major challenges facing the HIV prevention field. Presenting his long-term vision for control of the pandemic, Dr. Fauci introduced the image of two roads converging toward eventual control. The two roads represent vaccine-based and nonvaccine-based strategies, with the latter group including condom use, voluntary medical male circumcision (VMMC), prevention of mother-to-child transmission, treatment as prevention (TasP), and pre-exposure prophylaxis (PrEP). He emphasized that control of the pandemic appears to be a more realistic goal at this time, rather than complete eradication, and that a vaccine capable of reaching 50%–60% efficacy, used in combination with other prevention and treatment strategies, could help attain this goal. While the number of new HIV infections is declining globally, key populations such as adolescent girls and young women in southern Africa, female sex workers, men who have sex with men (MSM), the Lesbian Gay Bisexual Transgender Queer community and people who inject drugs are still disproportionately affected by the epidemic.^2^ These plenary talks set the stage for the remainder of the conference, which also focused on vaccine-based and nonvaccine-based strategies for HIV prevention. A list of HIV prevention clinical trials discussed at HIV Research for Prevention (HIVR4P) 2018 is presented in Table 1.

**Table 1.** HIV Prevention Clinical Trials that are Described in this Conference Summary

*Trial*	*Intervention*	*Phase*	*Strategy*	*Trial sites*	*Population*	*Status*
AMP STUDY	Antibody infusion	2b	Intravenous infusion of human mAb, VRC01	Botswana, Kenya, Malawi, Mozambique, South Africa, Tanzania, Zimbabwe	Women, ages 18–50	Ongoing
HVTN 703/HPTN 081						
AMP STUDY	Antibody infusion	2b	Intravenous infusion of human mAb, VRC01	Brazil, Peru, Switzerland, United States	Men (MSM) and transgender persons, ages 18–50	Ongoing
HVTN 704/HPTN 085						
ASPIRE (MTN 020)	Vaginal ring	3	DPV ring	Malawi, South Africa, Uganda, Zimbabwe	Women, ages 18–45	June 2015
CHAPS	PrEP	2	On-demand oral TAF/FTC compared with TDF (TDF/FTC) for insertive sex	South Africa, Uganda, Zimbabwe	Men, ages 13–24	Ongoing
DISCOVER	PrEP	3	Daily, oral TAF/FTC compared with TDF (TDF/FTC)	Austria, Canada, Denmark, France, Germany, Ireland, Italy, The Netherlands, Spain, United Kingdom, United States	Men (MSM) and TGW, ages ≥18	Ongoing
ECHO (Evidence for Contraceptive Options and HIV Outcomes) study	Hormonal contraceptives and HIV		DMPA, LNG	South Africa, Kenya, Swaziland, Zambia	Women, ages 16–35	Ongoing
HPTN 046	TasP	3	Oral Nevirapine	South Africa, Tanzania, Uganda, Zimbabwe	Pregnant women ≥18 and their infants	Completed November 2011
HPTN 077	PrEP	2a	Long-acting, injectable cabotegravir	Brazil, Malawi, South Africa, United States	Men and women, ages 18–65	Completed July 2018
HPTN 083	PrEP	3	Injectable cabotegravir LA for PrEP	Argentina, Brazil, India, Peru, South Africa, Thailand, Vietnam, United States	Men (MSM) and TGW, ages ≥18	Ongoing
HPTN 084	PrEP	3	Long-acting injectable cabotegravir compared with daily oral TDF/FTC	Botswana, Kenya, South Africa, Uganda, Zimbabwe	Women, ages 18–45	Ongoing
HVTN 097	Vaccine	1b	ALVAC and AIDSVAX B/E, tetanus toxoid vaccine and hepatitis B vaccine	South Africa	Women and men, ages 18–40	Completed February 2015
HVTN 100	Vaccine	1/2	ALVAC prime and bivalent subtype C gp120/MF59 boost	South Africa	Men and women, ages 18–40	Completed August 2018
HVTN 106	Vaccine	1	Nat-B/CON-S/Mosaic Env DNA prime, MVA-CDMR boost	Switzerland, United States	Women and men, ages 18–50	Ongoing
HVTN 111	Vaccine	1	Subtype C DNA prime and subtype C gp120/MF59 boost	South Africa, Tanzania, Zambia	Men and women, ages 18–40	Ongoing
HVTN 702	Vaccine	2b/3	ALVAC prime and bivalent subtype C gp120/MF59 boost	South Africa	Men and women, ages 18–35	Ongoing
HVTN 705	Vaccine	2b	Ad26.Mos4.HIV prime, Clade C gp140 boost	Malawi, Mozambique, South Africa, Zambia, Zimbabwe	Women, ages 18–35	Ongoing
MTN 030/IPM 041	Microbicides	1	DPV/LNG vaginal rings	United States	Women, ages 18–45	Completed August 2017
Promise study	TasP	3	Oral ZDV, Nevirapine Truvada, Lamivudine-ZDV, Lopinavir-ritonavir	India, Malawi, South Africa, Tanzania, Uganda, Zambia, Zimbabwe	Pregnant, postpartum women and their children	Completed September 2016
Quatro study	Vaginal delivery forms—gel, insert, film, or IVR		Placebo delivery through hydroxyethyl cellulose gel, vaginal insert, film, or IVR	South Africa, Zimbabwe	Women, ages 18–30	Completed September 2017
RING STUDY IPM 027	Microbicides	3	DPV vaginal ring	South Africa, Uganda	Women, ages 18–45	Completed December 2016
RV144	Vaccine	3	ALVAC prime and VaxGen gp120 B/E boost	Thailand	Men and women, ages 18–30	Completed June 2009
RV217	Early Capture HIV Cohort Study (ECHO)		Prospective natural history study following high-risk volunteers	Kenya, Tanzania, Thailand, Uganda	Men and women, ages 18–50, at high risk of HIV-1 infection	Completed September 2018
Tsepamo study	TasP	3	DTG, FTC/TAF or DTG, FTC/TDF or efavirenz/FTC/TDF	Botswana	Pregnant women, ages ≥18 and their infants	Ongoing
UChoose	Contraceptive methods as proxy for HIV prevention methods		Injectable Nur-Isterate, intravaginal Nuvaring and daily, oral Triphasil tablet	South Africa	Women, ages 16–17	Ongoing
W001	Vaccine	1	Recombinant BG505 SOSIP gp140 protein trimer	Kenya, United States	Men and women, ages 18–50	Ongoing

DMPA, depot medroxyprogesterone acetate; DPV, dapivirine; DTG, dolutegravir; EFV, efavirenz; FTC, emtricitabine; IVR, intravaginal ring; LNG, levonorgestrel; MSM, men who have sex with men; PrEP, pre-exposure prophylaxis; TAF, tenofovir alafenamide; TasP, treatment as prevention; TDF, tenofovir disoproxil fumarate; TGW, transgender women.

## More Signposts on the Road to HIV Vaccines

The journey of discovery toward an effective HIV vaccine may be longer than many imagined, but the consensus from this conference was that there are now more signposts than ever to guide the way. HIVR4P included a wealth of presentations exploring our growing knowledge of immune responses, updates from studies currently underway, views of strategies to improve the results of RV144, and an examination of the growing pipeline of vaccine approaches. These include broadly neutralizing antibodies (bNAbs) and CD8+ T-cell pathways to a vaccine, and DNA, RNA, and mosaic strategies. As concluded by **John Mascola** (Abstract PL04.04)^1^ in his final plenary talk, “*The path [to vaccine development] will not be short, it will probably be bumpy, but reaching the final goal is likely.*”

Current vaccine studies fall into three broad categories: (i) those following the RV144 trial and aimed at induction of durable antibody-dependent cell-mediated cytotoxicity (ADCC)-promoting antibodies; (ii) those aimed at eliciting bNAbs, either by mimicking Env vulnerability sites or by driving immune responses from germline B-cells; and (iii) those aiming to elicit robust T-cell responses, including those directed toward potentially protective but subdominant epitopes.

### RV144 follow-up

As described by **Linda-Gail Bekker** (Abstract PL01.01)^1^ in an Opening Plenary talk, the partial protection observed in RV144 has led to several subsequent vaccine trials. RV144 remains the only vaccine trial to demonstrate efficacy against HIV acquisition, with antibodies directed against the V1V2 region of gp120 and ADCC-mediating antibodies correlating with lower acquisition rates, and plasma envelope (Env)-specific IgA antibodies correlating with risk.^3,4^ HVTN 100 was a phase 1/2 study in low-risk, HIV-uninfected South Africans combining ALVAC-HIV and subtype C gp120 adjuvanted with MF59. As reported by **Xiaoying Shen** (Abstract OA02.03),^1^ HVTN 100 elicited anti-V1V2 binding antibody responses and antibodies capable of mediating phagocytosis and ADCC; however, V1V2 antibody magnitude and breadth were lower than those reported in RV144 and HVTN 097. A comparison of HVTN 100 and HVTN 111, presented by **Zoe Moodie** (Abstract OA02.04LB),^1^ revealed induction of higher anti-V1V2 IgG titers, NAb titers, and CD4+ T-cell responses with DNA prime compared with ALVAC prime, supporting further evaluation of DNA in prime-boost regimens with Env proteins. Early data from HVTN 100 paved the way for HVTN 702 (UHAMBO), a randomized, double-blind study that is currently recruiting participants, and will test immunogens optimized for prevention of Clade C infections.

### Novel immunogen design

In a comprehensive overview, **Rogier Sanders** (Abstract PL03.03)^1^ summarized current immunogen design efforts, outlining four hypotheses driving these efforts. The “native trimer” hypothesis predicts that a stable structural and antigenic mimic of the native, cleaved Env trimer will induce neutralizing antibodies. This is being tested with a third-generation native-like trimer, BG505 SOSIP.664, in the vaccine trial designated as W001. The second or “lineage immunogen” hypothesis is based on the observation that bNAbs do not emerge immediately in response to one antigen, but instead require coevolution of the virus and the antibody response. Therefore, sequential immunization with a series of Envs mimicking those longitudinally produced in individuals who develop bNAbs over time may induce similar antibodies. This is being tested by CHAVI-ID using sequential immunogens derived from CH505 gp120 and SOSIP trimers.

In a related approach, **José Alcami**, **Eloisa Yuste**, and colleagues (Abstract SY01.03)^1^ have synthesized Env trimers from very rare “acute neutralizers”; these will be tested in a European trial beginning in 2019 within the EAVI2020 project. The third or “germline-targeting” hypothesis predicts that Env immunogens engineered to target germline precursors will effectively prime bNAbs. Leading immunogens in this category are the eOD-GT8 60-mer described by **Bill Schief** (Abstract SY01.05),^1^ 426cTM, and BG505 GT1.1, all of which are planned for upcoming clinical trials. The fourth or “epitope-focusing” hypothesis reviewed by Dr. Sanders reasons that Env vaccines contain many potentially distracting epitopes; accordingly, priming with a highly focused immunogen might produce a more focused response. The leading immunogen in this category, the fusion peptide-based immunogen FP8_v1-rTTHC, will begin clinical trials in 2019 led by the NIH VRC.

### Mosaic immunogen approaches and T-cell-based vaccines

In HVTN 106, a phase 1 study reported by **Nicole Frahm** (Abstract OA07.01),^1^ a trivalent group M T-cell mosaic DNA vaccine was compared with DNA sequences from transmitted/founder clade B.1059, or group M centralized sequences (ConS). The magnitude of Env-specific CD4+ T-cell responses was higher in the group receiving the mosaic vaccine, and response breadth was also greater in the group primed with mosaic DNA. Current and future studies utilizing mosaic immunogens include the phase 2b HVTN 705 study (IMBOKODO), which will utilize Ad26.Mos4.HIV mosaic antigens combined with trimeric Clade C gp140 protein. This strategy has shown significant protection in nonhuman primates and is demonstrably immunogenic in humans. Talks by **Wendy Burgers** (Abstract SY06.02)^1^ and **Christian Brander** (Abstract SY06.03)^1^ reviewed the role of T-cells in vaccine-mediated protection. Studies to date suggest a role for CD4+ T-cells in supporting effective antibody development, and work from Louis Picker's laboratory on the cytomegalovirus-vectored simian immune deficiency vaccine in rhesus macaques indicates a critical role for vaccine-induced, nonclassical CD8+ T-cell responses, restricted by Major Histocompatibility Complex class II and class I histocompatibility antigen, alpha chain E. There remain gaps in our understanding of the role of T-cells in protection from and/or clearance of infection, as well as of how best to measure T-cell responses, and what tissues should be sampled. **Michael Gale** (Abstract SY08.04)^1^ presented transcriptome data from animals in the RhCMV/SIV vaccine study, showing clear segregation between protected and unprotected animals by 3 days postvaccination.

## Antibody-Mediated Prevention

Recombinant forms of bNAbs are known to be safe when administered by infusion to uninfected individuals, also known as passive immunization. **Lynn Morris** (Abstract SY01.02)^1^ provided an overview of ongoing and planned antibody-mediated prevention (AMP) studies. Two studies are currently underway to evaluate the efficacy of the VRC01 bNAb: HVTN 704/HPTN 085, which focuses on MSM and transgender individuals in the Americas, and HVTN 703/HPTN 081, which enrolled women in sub-Saharan Africa. As mentioned previously, there is also a pipeline of double and triple bNAb combinations, with improved breadth and potency toward acute-early Clade C viruses, which will be tested in future trials. The current AMP studies are seen as pivotal proof-of-concept trials; if successful, results of these trials will strengthen the rationale for bNAb-based vaccines, and pave the way for improved prevention trials with passive administration of bNAbs.

Importantly, AMP strategies can also be used to prevent mother-to-child transmission. **Ann Hessell** (Abstract OA13.06LB)^1^ extended her earlier findings that postexposure prophylaxis with passive infusion of bNAbs is able to protect newborn macaques from oral exposure to SHIV. Protection is time- and dose dependent, and is most effective when the bNAbs are present before the virus is widely disseminated throughout the body.

New data presented at HIVR4P described different improved AMP strategies currently under development, including those relying on vector-based delivery, such as adeno-associated virus-based strategies presented by **José Martínez-Navío** (Abstract OA13.05)^1^; or those modulating antibody interaction with neonatal Fc receptors to increase plasma half-life; antibody combinations with different specificities; and synthetic bi- or trispecific antibodies. The latter approach was exemplified by antibody mimetics such as newly designed ankyrin repeat proteins (DARPins) presented by **Matthias Glögl** (Abstract PD01.01)^1^ and a new engineered bispecific antibody, which successfully protected humanized mice from HIV acquisition. These antibodies, developed by **Zhiwei Chen** (Abstract SY01.01),^1^ recognize both gp120 and CD4.

## Advances in Basic Science

### HIV transmission and dissemination

New findings presented at HIVR4P focused on aspects of mucosal HIV transmission and early dissemination. **Thomas Hope** (Abstract PL02.01)^1^ described the use of novel imaging approaches to answer questions about early HIV transmission, mucosal reservoirs during antiretroviral therapy (ART), antibody distribution, and PrEP efficacy in an *in vivo* nonhuman primate model. His group has been at the forefront of developing and applying new technologies to localize and quantify viral reservoirs, particularly within mucosal tissues. In a session focused on Mucosal Models of Prevention, **Rosemary Bastian** (Abstract OA06.01)^1^ showed that binding affinity between HIV-specific IgG antibodies and various mucins correlated with *in vitro* enhancement of neutralization potency. **Marta Rodriguez-Garcia** (Abstract OA06.02)^1^ explored how neutrophils from the female genital tract capture HIV through release of extracellular traps.

**Manish Sagar** (Abstract SY02.01)^1^ identified a vaginal resident CD1a^+^ classical dendritic cell (DC) population able to support CCR5-tropic but not CXCR4-tropic HIV-1 replication *in vitro*, suggesting that these cells might play an active role in the selection of transmitted viral variants during sexual HIV acquisition. **Andrew Harman** (Abstract SY02.02)^1^ provided evidence of CD11c+ DCs highly susceptible to CCR5-tropic HIV-1 in male and female genital mucosa. Data from **Eric Arts** (Abstract SY02.03)^1^ supported a role of migrating DC selecting and disseminating virus from mucosal sites. He also provided evidence for selection of specific patterns of glycosylated viruses, raising the question of whether active recruitment/infection of lectin-expressing DC could be a mechanism for preferential transmission of viral variants. Finally, **Alexandra Schuetz** (Abstract SY02.04)^1^ provided new data on the impact of early treatment on HIV replication and mucosal T-cells in an acute infection cohort from Thailand. Patients treated beginning at Fiebig stage I had lower levels of immune activation, but interruption of ART led to viral rebound in patients treated at all stages.

Reporting on Lake Victoria fishing communities with a high prevalence of both HIV and *Schistosoma mansoni*, **Rupert Kaul** (Abstract OA17.02LB)^1^ showed that schistosome treatment with Praziquantel reduced HIV entry into cervical and blood CD4^+^ T-cells. **Morgane Rolland** (Abstract OA17.03)^1^ used phylodynamic analysis and modeling of viral growth rates to more accurately assess the duration of the eclipse phase of HIV infection. In the RV217 HIV-1 acute infection cohort, the eclipse phase was estimated to last 6 days for infections by single founders, with no obvious effect linked to virus subtype or gender. In oral transmission studies using SIVmac251 to model neonatal transmission in rhesus macaques, **Roslyn Taylor** (Abstract OA17.05)^1^ found that the entire digestive tract is susceptible to replicative viral infection, and that the majority of infected cells are T-cells. **Nicole Naiman** (Abstract OA17.06)^1^ presented data from mother–infant pairs collected in Nairobi during the 1990s in a breastfeeding study. IgG binding to the gp41 ectodomain was associated with increased risk of mother-to-child transmission.

### A role for the microbiome in HIV susceptibility?

Finally, several speakers discussed the role of microbial diversity in HIV susceptibility. **Heather Jaspan** (Abstract SY10.01)^1^ reported that higher diversity was consistently associated with increased HIV risk, as were inflammation and recruitment of target cells to the mucosa. **Connie Celum** (Abstract SY10.02)^1^ reported on increasing rates of other sexually transmitted pathogens, notably *Chlamydia trachomatis* and *Neisseria gonorrhoeae* infection in North America, especially in MSM, and emphasized the need for more innovative ways to address this problem. **Cara Wilson** (Abstract SY10.04)^1^ reported on the use of *in vitro* systems to model the complex interactions between immune cells, HIV, and intestinal microbiota. Her group found that mucosal butyrate-producing bacteria were associated with reduced CD4+ T-cell proliferation and activation.

Mucosal microbiomes influence susceptibility to disease and response to treatment. There is strong evidence that vaginal dysbiosis increases risk of HIV acquisition. However, the relationship between hormonal contraception and HIV susceptibility is less clear. Studies of multiple contraception methods have found that hormonal contraception optimizes vaginal bacteria over time (as measured by decreased Nugent score). **Sharon Achilles** (Abstract PL02.03)^1^ argued that these findings rule out dysbiosis as a pathway for hormonal contraception to increase HIV risk. Two other possible mechanisms are changes in bleeding patterns and decreased condom use. For the vaginal ring (NuvaRing), 3 months of data demonstrated a decrease in dysbiosis, though accumulation of biomass on the ring was positively associated with dysbiosis. Considering these findings, **Dr. Achilles** identified priority research questions: What happens to the vaginal microbiome when women have more bleeding, even if it is light spotting (as occurs in some women after starting hormonal contraception)? What happens to microbiome with vaginally delivered drugs? What are the longitudinal impacts of intrauterine devices (IUDs) or implants on the vaginal microbiome, and how does the microbiome alter the metabolism and efficacy of these devices?

### Contraception, pregnancy and HIV risk

Hormonal contraception products such as injectable depot medroxyprogesterone acetate (DMPA) and norethisterone enanthate (NET-EN) may modulate HIV acquisition risk; however, the biological mechanisms at play remain incompletely understood. **Chanel Avenant** (Abstract OA12.01)^1^ showed that both MPA and luteal phase hormones increased HIV-1 infection of peripheral blood mononuclear cell and TZM-bl cells in the laboratory. In the case of MPA, the mechanism involved increased activation of CD4+ T-cells and increased expression of CCR5, mediated through the glucocorticoid receptor. In a related study, **Taguma Matubu** (Abstract OA12.04)^1^ reported that DMPA, but not NET-EN, decreased T-cell activation in response to polyclonal stimulation, suggesting a possible window of immune suppression at peak MPA levels. This “window” could translate to heightened susceptibility to HIV infection. The ongoing ECHO trial of DMPA, the Jadelle implant, and the copper IUD to evaluate whether any of the three methods might actually increase the risk of HIV acquisition was described at the conference, and results are expected in mid-2019. A phase 1 pharmacokinetic (PK) study of a dapivirine (DPV) and levonorgestrel (LNG) vaginal ring for combined prevention of HIV and pregnancy risk [MTN-030/IPM 041, presented by **Sharon Achilles** (Abstract OA12.02LB)^1^] demonstrated that this ring was safe, well tolerated, and resulted in adequate DPV and LNG exposure.

Focusing on “safe conception” strategies for couples living with HIV, **Renee Heffron** (Abstract OA12.05)^1^ reported on a pilot study of HIV prevention in the context of pregnancy optimization for HIV serodiscordant couples in Kenya. Couples utilized a variety of safe sex strategies, including ART, PrEP, VMMC, or timed condomless sex acts. Encouragingly, the study detected no new HIV infections among the 74 couples enrolled.

## PrEP Comes of Age

PrEP impact, PrEP access, and the possibility that PrEP works differently for different people were running themes throughout HIVR4P 2018. Conference sessions and presentations looked at the current and potential future impact of PrEP, scale-up options, and opportunities and challenges of building social, political, and financial support to make PrEP available for those who could benefit most. Abstracts focused on PrEP acceptability, mathematical modeling of PrEP targeting, costs and benefits, and a host of presentations on emerging PrEP drugs and delivery options that could greatly increase the future impact of PrEP were also discussed.

**Jared Baeten** (Abstract OA23.01)^1^ reported on HIV incidence in persons using Truvada [emtricitabine (FTC)/tenofovir disoproxil fumarate (TDF)] in a total of 46 PrEP demonstration projects worldwide, involving >10,000 participants. Encouragingly, only 91 incident infections were detected, of which 27 occurred >30 days after the final dose of Truvada was taken and 17 occurred within the first 3 months of initiating PrEP, thus may have resulted from an earlier exposure. This translates to an incidence rate of <1% per year. These findings emphasize that PrEP adherence is critical for success.

### Who is using PrEP?

**Laura Fitch** (Abstract OA04.01)^1^ presented data from AVAC's Global PrEP tracker (PrEPWatch.org). Data from the first quarter of 2018 indicated that 309,525 people have initiated PrEP globally, with the highest numbers in North America (71%) and sub-Saharan Africa (15%). In North America, the majority of users are MSM, while the majority of users in sub-Saharan Africa are adolescent girls and young women. Significant challenges to PrEP uptake and continuation remain, even in areas of relatively high current usage. **Albert Liu** (Abstract OA04.06)^1^ reported on PrEP awareness among HIV-uninfected MSM and transgender women (TGW) in San Francisco. In this study, 97% of MSM and 79% of TGW were aware of PrEP, yet only 40% of MSM and 15% of TGW had used PrEP despite high levels of health insurance coverage and engagement in health care. **Irene Mukui** (Abstract SY04.03)^1^ described progress and lessons learned from the national rollout of PrEP in Kenya. As of May 2017, only 1,425 Kenyans were taking PrEP compared with 17,466 in August 2018. This impressive increase was achieved through coordinated efforts of national and international stakeholders, community members, and a focus on specific geographic areas with high incidence clusters.

### Optimizing PrEP delivery

**Renee Heffron** (Abstract SY07.01)^1^ provided an overview of programs that incorporate oral PrEP into programs addressing each stage of women's reproductive health. For those who desire pregnancy, PrEP can be provided efficiently through safer conception programs. Additional opportunities include integration of HIV prevention into antenatal care and prevention of mother-to-child transmission, family planning clinics and other places where emergency contraception or abortion care is provided. Challenges to integration were discussed, including low funding levels, nonsupportive policies, and low provider comfort. **Nittaya Phanuphak** (Abstract SY07.04)^1^ described the successes of PrEP programs led by key populations in Thailand. An impressive scale-up to 5,000 persons currently on PrEP, up from zero in 2014, was achieved primarily due to community involvement in program planning and implementation.

In a captivating presentation, **Sarit Golub** (Abstract SY07.03)^1^ identified flaws in current risk-assessment practices that contribute to stigma and alienation of potential PrEP users. She described an alternative approach to discussing prevention, sexual health and HIV prevention options, counseling, in which clients and providers work collaboratively to understand and address individual clients' personal health priorities. This approach focuses on reasons why people might want to take PrEP rather than focusing on “risk,” thereby reducing anxiety, increasing sexual satisfaction, and improving the sense of control over one's own sexual health.

### New ARVs and combination products for prevention

**Raphael Landovitz** (Abstract PL03.01)^1^ provided a comprehensive overview of work to identify new systemic PrEP products, including oral tenofovir alafenamide (TAF)/FTC, injectable cabotegravir, bNAbs, the DPV vaginal ring, implantable devices, and microneedle array patches. In the DISCOVER trial, the safety and efficacy of daily oral TAF/FTC are being compared with TDF/FTC. Trials are also underway to evaluate the safety and efficacy of long-acting injectable cabotegravir, which is being compared with daily oral TDF/FTC in HPTN 083 and 084. In AMP trials, discussed elsewhere, the safety and efficacy of two different doses of bimonthly passively infused VRC01 bNAb are being compared with placebo. The ASPIRE and Ring trials previously demonstrated an ∼30% risk reduction conferred by the DPV vaginal ring; subsequent open-label studies pointed to greater, ∼50%, risk reduction. This product is currently under regulatory evaluation. Finally, other novel approaches under development include implants and microneedles, which puncture the stratum corneum of the skin, allowing direct delivery of drug into underlying tissues. These can take several forms, including dissolving patches applied to the skin. The range of products currently under development provides hope that the future of biomedical HIV prevention will involve user-empowering choices.

In an oral abstract session focused on the development of new ARVs, **James Cummins** (Abstract OA15.01)^1^ provided data on the PK-pharmacodynamic (PK-PD) properties of drug-containing intravaginal rings (IVRs) containing the second-generation integrase inhibitor MK-2048 alone or in combination with vicriviroc, inserted and retained for 14 days in Rhesus macaques. Results indicated the utility of the macaque model for evaluating PK of MK-2048 and confirming its antiviral activity in vaginal tissues.

## Treatment as Prevention

### Undetectable equals untransmittable

**Nelly Mugo** (Abstract RT02.02)^1^ reviewed the science of “undetectable = untransmittable” (U = U), the observation that suppressive ART has been convincingly and repeatedly demonstrated to prevent sexual transmission of HIV. Based upon multiple published studies, the World Health Organization now states that there is scientific “consensus that people who have achieved and maintained an undetectable viral load cannot transmit HIV sexually to their HIV negative partners.” This is true irrespective of sexual practices (anal, vaginal, and penile condomless penetrative sex), and within the context of high transmission rates of other sexually transmitted infection. However, she also stressed that community trials with weak linkage to ART treatment after a HIV diagnosis have failed to produce similar reductions in HIV incidence. **Bruce Richman** (Abstract RT02.05)^1^ emphasized the transformative nature of U = U in dismantling HIV stigma and providing a strong argument for universal health care access. He emphasized that this message is difficult to communicate to the public at large, because “science doesn't have a publicist,” and there are many layers of long-standing stigma to overcome.

### Breastfeeding is an exception

**Lena Serghides** (Abstract RT02.03)^1^ reported that mother-to-child transmission through breastfeeding appears to be an exception to the rule of U = U. In the PROMISE trial of >2,000 infants and their mothers, transmission rates of 0.6% and 0.9% were seen within 12 and 24 months of breastfeeding, respectively. Two of eight women who transmitted the virus to their infants had confirmed undetectable plasma viral load. Similarly, in the HPTN 046 trial, transmission among women on ART was 0.5% at 18 months. Although cell-free virus is largely eliminated by ART, latently infected CD4+ T-cells harboring cell-associated HIV DNA are not. Given the large amount of breastmilk consumed over a 6-month period, infants may be exposed to many latently infected CD4+ T-cells. Furthermore, some women have detectable HIV RNA in breastmilk despite undetectable levels in plasma. Thus, unfortunately U does not equal U in the context of mother-to-child transmission. That said, breastfeeding decisions in resource-poor settings must also weigh the importance of preventing malnutrition and diarrheal/pneumonia diseases against the relatively low risk of HIV transmission for women on suppressive cART.

## The Future of Prevention Research

If a single sentiment permeated this third biennial HIVR4P meeting, it may have been the sense of excitement at the richness of the prevention pipeline, and the variety of new prevention approaches and products in development. Presentations on new prevention drugs and delivery systems—including inserts, implantables, gels, biodegradable polymers, refillable reservoirs, osmotic pumps, and more—drew crowds in Madrid. So did discussions about the opportunities and obstacles created by the increasing number of prevention options available and in development. Among these are the challenges of evolving trial design, the need to understand user preference and evaluate health system capacity, managing product introduction and, of course, maintaining financial and political support for biomedical HIV prevention.

### Trial design in the PrEP era

A major challenge for clinical trial design in the PrEP era is that alternative PrEP agents are being compared with oral TDF/FTC as the current standard of care. **Sheena McCormack** (Abstract PL01.02)^1^ discussed novel trial designs to assess the efficacy of new HIV prevention products in this environment. Noninferiority studies require a larger sample size and longer follow-up than traditional studies due to the high efficacy of the TDF/FTC regimen. To overcome this challenge, background HIV incidence in the source population may be used as an estimate of what incidence would be in a hypothetical placebo group. This can be incorporated into a novel measure of effectiveness, termed the averted infections ratio, which compares the proportion of infections averted by the new drug with that averted by TDF/FTC. Another approach, also addressed by **Deborah Donnell** (Abstract SY03.02),^1^ uses a multiarm, multistage trial design, allowing multiple agents and/or interventions to be compared with a single placebo group, thereby reducing the number allocated to placebo. From the regulatory perspective, **Jeffrey Murray** (US FDA) (Abstract SY03.01)^1^ noted that noninferiority trials in women may be difficult due to the absence of consistent historical comparison data.

### HIV prevention in women of childbearing age

**Anne Lyerly** (Abstract SY11.01)^1^ explored ethical considerations regarding HIV prevention trials in women of childbearing age. She showed how exclusion of pregnant women from research studies means that knowledge about the PDs and safety in pregnancy of medications used for HIV treatment and prevention lags many years behind drug approval.

**Elaine Abrams** (Abstract SY11.02)^1^ discussed recent findings from the Tsepamo study that Dolutegravir (DTG) use at conception may increase the risk of neural tube defects, leading to WHO guidelines restricting DTG use among women of childbearing age to those using reliable contraception. She drew parallels with historical restrictions on the use of Efavirenz (EFV) by women of childbearing age, based upon small numbers of cases of neural tube defects, which hampered treatment rollout. After more than two decades, she noted, EFV appears to be safe and is not associated with neural tube defects. **Sharon Hillier** (Abstract SY11.04)^1^ asked “How much do any of us really understand about risk?” She highlighted that while overall there had been a balanced response to warnings regarding DTG use among women of childbearing age, there are lessons that can be learned. She discussed that after WHO warnings about DTG use in pregnancy, the HPTN 084 study, examining the efficacy of long-acting cabotegravir (CAB LA)—structurally related to DTG—among women aged 18–45 has required its participants to use long-acting contraception, meaning that there will be data gaps on the safety of yet another PrEP drug in pregnancy and periconception.

## Choice in Prevention Options

### “Show Me The Ring”

The themes of user choice and the need for more and better user-controlled prevention options, including options that can protect women without their partner's knowledge, resonated throughout the conference. Interest was high in the status of the DPV ring, as well as in new rings and other female-controlled products in development. Updates on vaginal and rectal microbicides also filled conference rooms in Madrid, with delegates anxious to learn the latest on these long-sought-after forms of prevention.

**Craig Hendrix** (Abstract PL03.02)^1^ reviewed the pipeline for on-demand, topical (vaginal/rectal) PrEP. He summarized evidence that such products can be effective, arguing that choice from among a range of prevention options leads to increased uptake, drawing parallels with contraception. He described vaginal and anal microbicides in development, beginning with the Pod-IVR, a vaginal ring that can deliver flexible drug combinations. Studies published in 2018 revealed that some drugs (FTC and MVC) can achieve protective levels in the rectum when delivered through pod vaginal rings. Other novel products included fast-dissolving vaginal films, vaginal and rectal inserts, and numerous multipurpose formulations that provide protection against other STIs and/or deliver contraceptive agents. At least eight phase 1 trials of rectal microbicides have been or will soon be conducted. Several of these are aiming to be behaviorally congruent; that is, by adding PrEP to a product that people already use as part of their sexual routine, in this case lubricants and douches used by >85% and >75% of MSM, respectively. Studies have shown promising feasibility and acceptability of a rectal microbicide douche for MSM. **Dr. Hendrix** finished with a call to ensure that microbicide development continues to be a priority, to provide as wide a range of HIV prevention choices as possible.

Presented by **Elizabeth Montgomery** (Abstract OA04.04),^1^ the Quatro study of 200 young women in Zimbabwe and South Africa tested the acceptability of four vaginal placebo delivery modalities: monthly rings and precoital films, inserts and gels. The findings from adherence marker evaluation confirmed that most women did use the products, over half of the women who chose an on-demand product used them with sex, and older women were more adherent. Findings from the study highlighted that users desired multiple prevention options; accordingly, women should be given various options to choose from.

Adolescents in South Africa account for 40% of new infections, yet little is known about preferred prevention modalities in this group. Findings from UChoose, a study of prevention choices in adolescent women ages 15–19, were presented by **Katherine Gill** (Abstract OA05.06LB).^1^ This open-label, randomized crossover study used licensed contraceptive modalities (monthly vaginal rings; injectables; oral contraceptives) to assess acceptability and preferences. Results suggested that long-acting injectable PrEP would have the highest acceptability, followed by vaginal rings, and finally, oral pills.

### “Give Me Prevention That Lasts”

Continuing with the theme of choice in prevention, those seeking news on systemic prevention approaches encountered an expanding universe of data on long-acting PrEP at HIVR4P, including the first tail-phase data on long-acting injectable cabotegravir in women.

Reported by **Elizabeth Tolley** (Abstract OA05.01),^1^ A phase 2 study by the HIV Prevention Trials Network, HPTN 077, sought to test acceptability of CAB LA in HIV uninfected, low-risk men and women in Brazil, Malawi, South Africa, and the United States. There was a strong preference for long-acting injectable PrEP at both baseline and follow-up, particularly from participants outside the United States.

**Susan Ford** (Abstract OA15.05)^1^ presented a CAB Population PKs model that included intrinsic and extrinsic factors, developed with data from phase 1/2 studies and simulations that can be used to inform PrEP phase 3 dosing strategies. Results revealed that the CAB LA absorption rate differs between males and females, and provided information that can be used to develop recommendations for reinitiation strategies for those with prolonged interruptions in dosing.

Finally, **Raphael Landovitz** (Abstract OA15.06LB)^1^ and colleagues provided results from HPTN 077 evaluating tail-phase safety, tolerability, and PKs of long-acting injectable CAB in HIV-uninfected individuals. The apparent half-life of CAB LA for females was significantly longer compared with males, and was also influenced by body mass index. The median time to the lower limit of quantification (LLOQ) was 66.3 weeks for females and 42.7 weeks for males. At 76 weeks after the final injection, 58% of females and 87% of males had levels below the LLOQ.

## Concluding Messages

In the two years since the HIVR4P 2016 conference, consistent progress has been made toward improving and expanding HIV prevention strategies. As prevention science advances, however, the challenges of maintaining the political will and funding to support a global movement to end the AIDS epidemic come into even sharper focus.

New strategies of vaccine design are following several well-defined routes to reach 50%–60% efficacy, suggested as the lowest acceptable threshold for a vaccine candidate to be used in combination with other prevention strategies. In parallel, bNAbs are emerging as important new tools in the repertoire of feasible prevention strategies. For both these approaches, data from ongoing studies will be eagerly awaited at the next HIVR4P meeting.

Condom use, VMMC, prevention of mother-to-child transmission, TasP, and PrEP have also made significant strides as nonvaccine-based strategies for HIV prevention. Multipurpose products, providing protection against HIV and pregnancy, will provide exciting new options for women.

It was clear from discussions in the meeting rooms, poster sessions, and hallways that hope persists that the future of biomedical HIV prevention will include a wide array of prevention modalities. Behavioral research has demonstrated that preferences for specific modalities vary greatly, and the availability of multiple options may help us to develop a wider range of implementation strategies to reach more men and women worldwide. Ensuring that scientific advances progress from the laboratory to clinical trials to the community remains a challenge that HIVR4P participants are poised to address.

The next HIVR4P conference will be held in Cape Town, South Africa, in October 2020.

## References

[1] Abstracts of the HIV Research for Prevention Meeting HIVR4P, 21–25 October, 2018, Madrid. AIDS Res Hum Retroviruses 2018;34(S1):1–408 29140723 10.1089/aid.2016.5000.abstracts

[2] UNAIDS: Fact Sheet—World AIDS Day 2018, 2017 Global HIV Statistics. 6 pp. Available at www.unaids.org/sites/default/files/media_asset/UNAIDS_FactSheet_en.pdf (2018), accessed 312, 2019

[3] Corey L, Gilbert PB, Tomaras GD, Haynes BF, Pantaleo G, Fauci AS: Immune correlates of vaccine protection against HIV-1 acquisition. Sci Transl Med 2015;7:310rv317 10.1126/scitranslmed.aac7732 PMC4751141 26491081

[4] Kim JH, Excler JL, Michael NL: Lessons from the RV144 Thai phase III HIV-1 vaccine trial and the search for correlates of protection. Annu Rev Med 2015;66:423–437 25341006 10.1146/annurev-med-052912-123749

